# 70 Comparative study between PIMS and Kawasaki disease

**DOI:** 10.1093/rheumatology/keac496.066

**Published:** 2022-10-07

**Authors:** M Jalal, A Sakhi, G. Benbrahim Ansari, H Aboufaris, K Bouayed, S Ameayou, S Hassoun, S Nani

**Affiliations:** 1 Department of Pediatric Internal Medicine and Rheumatology, Hôpital Mère-Enfant A. Harouchi, Casablanca, Morocco; 2 Epidemiology Department, Faculty of Medicine and Pharmacy, Casablanca, Morocco

## Abstract

**Background:**

Kawasaki disease (kDa) is a multisystemic vasculitis affecting medium and small vessels with a predilection for the coronary arteries. It appears to be very similar to the pediatric multisystemic inflammatory syndrome (PIMS), which is a post-infectious inflammatory condition occurring after SARS-COV2 infection. Although these two entities have clinical and biological similarities, marked differences are identified. The aim of this study is to compare the two conditions in order to highlight the specific criteria of each of these related entities and to describe their demographic, clinical, biological and therapeutic characteristics in our context.

**Method:**

Single center prospective observational study from January 2021 to March 2022 was conducted, comparing children admitted for PIMS on the basis of persistent fever, inflammatory syndrome, and positive COVID-19 IgG serology to patients hospitalized for kDa according to American Heart Association criteria.

**Results:**

42 cases were recruited, among them 24 PIMS and 18 kDa, with a male predominance. The average age was 5 years for PIMS and 2 years and 7 months for kDa. All had a persistant fever with an average duration of 8 days as reason for consultation. Conjunctivitis was found in 88% of kDa *vs* 75% of PIMS and cheilitis in 94% of kDa *vs* 75% of PIMS. Skin rash, extremity involvement, cervical adenopathy were reported in both pathologies with respective percentages of 62.5%, 17.4% and 25% for PIMS *vs* 61.1%, 33.3% and 33.3% for kDa. Abdominal pain was reported in 54.2% of PIMS patients *vs* 5.6% of kDa. All patients had an inflammatory syndrome. The mean sedimentation rate was 67 mm at the first h in PIMS and 99 mm at the first h in kDa. The mean CRP was 147 mg/l in PIMS *vs* 132 mg/l in kDa. Lymphopenia was predominant in PIMS with 33% *vs* 11% in kDa. Cardiac enzymes were higher in PIMS with 29% of myocarditis and 15% of coronary dilatation *vs* 16.7% of coronary involvement in kDa. All PIMS patients received immunoglobulin infusion IV-IG, corticosteroid therapy, and an antiplatelet agent, whereas patients with kDa received IV-IG and acetyl salicylic acid in anti-inflammatory doses. Apyrexia was obtained at day 1 of treatment in the majority of patients, whereas 5.6% of kDa had persistent aneurysm.

**Conclusion:**

PIMS cases predominated over kDa cases during this pandemic period. The distinction between the two entities could be difficult given the clinico-biological similarities. Abdominal pain was significantly more frequent in PIMS patients, whereas lymphopenia and myocarditis were not. The prognosis was better in PIMS.

